# Effects of *Lacticaseibacillus rhamnosus* KF7-Fermented Milk on Cognitive Function, Gut Microbiota, and Fecal Metabolism in APP/PS1 Mice

**DOI:** 10.3390/foods15183304

**Published:** 2026-09-18

**Authors:** Yilin Zhang, Zhenmin Liu, Chunping You

**Affiliations:** State Key Laboratory of Dairy Biotechnology, Key Laboratory of Functional Dairy Products Processing, Ministry of Agriculture and Rural Affairs, Shanghai Engineering Research Center of Dairy Biotechnology, Dairy Research Institute, Bright Dairy & Food Co., Ltd., Shanghai 201103, China

**Keywords:** *Lacticaseibacillus rhamnosus* KF7, fermented skim milk, gut microbiota, gut–brain axis, cognitive impairment

## Abstract

Functional fermented dairy products are increasingly investigated as food-based approaches to support health, but their potential relevance to cognitive dysfunction remains unclear. In this study, we evaluated skim milk fermented with *Lacticaseibacillus rhamnosus* KF7 (KF7-fermented milk) in APP/PS1 transgenic mice. KF7-fermented milk was associated with better cognitive performance and lower amyloid-β plaque burden in APP/PS1 mice. The intervention was also associated with higher whole-blood 5-hydroxytryptamine, lower cerebral glutamate, lower whole-blood expression of pro-inflammatory cytokines, and less pronounced cortical microglial and astrocytic activation. In the intestine, KF7-fermented milk was associated with higher expression of selected barrier-related genes. Microbiome analysis showed differences in gut microbial composition, while fecal metabolomics identified secondary bile acid biosynthesis as the strongest FDR-supported pathway associated with the intervention. Overall, KF7-fermented milk was associated with differences in cognitive, pathological, inflammatory, intestinal, microbial, and metabolic features in APP/PS1 mice. These findings provide preclinical evidence supporting further investigation of KF7-fermented milk as a food-based intervention relevant to gut–brain health.

## 1. Introduction

With the rapid aging of the global population, age-related cognitive decline has become an important public health challenge. The prevalence of Alzheimer’s disease (AD), one of the most common neurodegenerative disorders, is increasing worldwide and places a substantial economic and social burden on patients, families, and healthcare systems [1]. Pathologically, AD is characterized by extracellular amyloid-beta (Aβ) plaque deposition, intracellular neurofibrillary tangles, and progressive loss of neurons and synapses, ultimately leading to impairment of learning and memory [2,3,4]. In addition to these hallmark changes, chronic neuroinflammation is increasingly recognized as an important contributor to disease progression, as reflected by elevated pro-inflammatory mediators and the activation of microglia and astrocytes around Aβ plaques [5]. Although several treatments can temporarily relieve symptoms, effective strategies to delay or reduce AD progression remain limited. In this context, increasing attention has been paid to diet and food components as modifiable factors that may influence cognitive health [6,7].

Among emerging research directions, the gut microbiota has attracted considerable interest in relation to AD. Altered gut microbial composition has been reported in both patients with AD and AD animal models, although the specific microbial signatures vary across studies [8]. For instance, reduced microbial diversity and shifts in dominant bacterial taxa have been observed in individuals with AD [9,10]. In APP_SWE_/PS1_ΔE9_ mice, early-life antibiotic treatment changed gut microbiota and was accompanied by lower Aβ deposition and reduced activation of plaque-associated microglia and astrocytes [11]. These observations support the concept that the gut microbiota is involved in AD-related pathology. Importantly, microbiota-derived metabolites are increasingly recognized as key mediators of gut–brain communication. Among them, bile acids and neurotransmitter-related metabolites are of particular interest because they participate in intestinal homeostasis, immune regulation, and neural signaling, and their dysregulation has been associated with neuroinflammation and cognitive impairment [12]. Together, these findings suggest that modulation of the gut microbiota and its metabolic outputs may offer a useful framework for developing food-based strategies to mitigate cognitive decline.

Probiotics, defined as live microorganisms that confer a health benefit on the host when administered in adequate amounts, have been explored for their potential effects on intestinal and systemic health. In recent years, some probiotic strains have also shown promise in the regulation of neuroinflammation, behavior, and cognitive function. In AD-related studies, strains from the genera *Lactobacillus*, *Bifidobacterium*, *Akkermansia*, and *Clostridium* have been reported to improve behavioral performance, reduce inflammatory responses, or modulate gut microbial imbalance [13]. Among them, *Lacticaseibacillus rhamnosus* is one of the most extensively studied probiotic species and has been associated with beneficial effects in intestinal disorders, immune dysfunction, metabolic disease, and mild cognitive impairment [14,15,16]. *L. rhamnosus* KF7 was isolated from kefir, a traditional fermented milk from Yunnan Province, China. In our previous work, KF7 showed favorable safety-related and functional characteristics, including extension of the mean lifespan of *Caenorhabditis elegans* [17]. We also found that live KF7 enhanced intestinal epithelial barrier function in a dual-environment in vitro co-culture model [18], while its fermentation supernatant exhibited strong monoamine oxidase inhibitory activity in vitro [19]. These observations suggest that KF7 may influence both gut barrier function and neuroactive metabolism, supporting its further evaluation in food-based interventions.

Fermented milk is more than a vehicle for probiotic delivery, as the dairy matrix contains viable microorganisms, milk-derived nutrients, and fermentation-generated compounds that may jointly influence host physiology. Given the widespread consumption of fermented dairy products, such products are of particular interest for the development of foods with potential health-promoting properties. Nevertheless, the potential relevance of probiotic-fermented milk to cognitive dysfunction and AD-like pathology remains insufficiently studied. In the present study, we evaluated *Lacticaseibacillus rhamnosus* KF7-fermented milk as a whole-product dietary intervention in APP/PS1 mice. Cognitive performance, Aβ deposition, inflammatory responses, and intestinal barrier-related changes were assessed, together with gut microbial composition and fecal metabolic profiles. The study aimed to characterize the biological changes associated with KF7-fermented milk and to provide preclinical evidence for further investigation of fermented dairy interventions in the context of gut–brain health.

## 2. Materials and Methods

### 2.1. Preparation of L. rhamnosus KF7-Fermented Milk

The preparation of *L. rhamnosus* KF7-fermented milk was adapted from the fermentation conditions used in our previous studies [18,19,20], with minor modifications for the present animal intervention. *L. rhamnosus* KF7 (CGMCC No. 6430) glycerol stock was supplied by the Dairy Research Institute of Bright Dairy & Food Co., Ltd. (Shanghai, China). The bacterial strain was streaked onto MRS agar plates (1106610500, GranuCult^®^, Merck, Darmstadt, Germany) containing 1.5% (*w*/*v*) agar and incubated anaerobically at 37 °C for 48 h. After three successive transfers, a single colony was inoculated into sterilized 10% (*w*/*v*) skim milk (A600669-0250, Sangon Biotech, Shanghai, China) and incubated at 37 °C for 20 h to obtain KF7-fermented milk. At the end of fermentation, the pH of the fermented milk was 4.7–4.8, and the viable bacterial count was 6.6 × 10^8^ CFU/mL. KF7-fermented milk was freshly prepared at regular intervals, stored at 4 °C, and equilibrated to room temperature before administration.

The fermentation conditions were selected based on our previous work and preliminary optimization experiments. Under comparable conditions, KF7-fermented milk or its supernatant exhibited monoamine oxidase inhibitory activity in vitro [19] and showed protective effects against lipopolysaccharide-induced barrier injury in Caco-2 monolayers [20]. Therefore, these established conditions were used to prepare the fermented milk product for evaluation in APP/PS1 mice.

### 2.2. Animals and Experimental Design

Six-month-old male APP_swe_/PS1_ΔE9_ transgenic mice (APP/PS1) and control C57BL/6J wild-type mice were purchased from Cyagen (Suzhou) Biotechnology Co., Ltd. (Suzhou, China). All mice were housed in a specific-pathogen-free facility maintained at 22 ± 2 °C and 40–60% relative humidity under a 12-h light/12-h dark cycle. Mice were fed a standard chow diet with *ad libitum* access to water.

After 1 week of acclimatization, mice were first stratified according to genotype into wild-type and APP/PS1 blocks. Within each genotype block, each mouse was assigned a random number generated using Microsoft Excel. The mice were then ranked according to the generated random numbers and allocated to the PBS or KF7 treatment groups. Accordingly, the 30 mice (16 wild type mice and 14 APP/PS1 transgenic mice) were assigned to four groups: WT-PBS (wild type mice receiving sterile PBS solution, n = 8), WT-KF7 (wild type mice receiving *L. rhamnosus* KF7-fermented milk, n = 8), Tg-PBS (transgenic mice receiving sterile PBS solution, n = 6) and Tg-KF7 (APP/PS1 transgenic mice receiving *L. rhamnosus* KF7-fermented milk, n = 8). Mice received 200 μL of the assigned treatment once daily by oral gavage for 60 consecutive days. The intervention consisted of KF7-fermented milk as a whole fermented product rather than an isolated KF7 cell suspension. Based on the viable count of the fermented milk (6.6 × 10^8^ CFU/mL), each 200-μL administration contained approximately 1.3 × 10^8^ CFU of viable *L. rhamnosus* KF7 cells. This daily dose was comparable to that used in a previous probiotic intervention study in APP/PS1 mice [21], and the selected volume was suitable for repeated daily oral administration throughout the 60-day intervention. The same volume of PBS was administered once daily to the corresponding control groups to ensure consistent handling and gavage conditions across groups. Food and water were available *ad libitum* throughout the intervention. Behavioral testing was performed after completion of the intervention.

The study protocol was designed to minimize the number of animals used and their suffering and was approved by the Institutional Animal Care and Use Committee (IACUC) of Shanghai Rat & Mouse Biotech Co., Ltd. (SHRM) (Shanghai, China) (permission number: 202202(24)).

### 2.3. Morris Water Maze (MWM) Test

Spatial learning and memory were evaluated using a Morris water maze (MWM) procedure adapted from the method originally described by Morris and subsequently standardized for rodent behavioral studies [22,23]. The test was performed in a circular pool (1.2 m in diameter) filled with opaque white water maintained at 25 ± 1 °C. Animal movement was recorded and quantified using SuperMaze animal behavior analysis software (version 3.3.0.0, Shanghai Xinruan Information Technology Co., Ltd., Shanghai, China). During behavioral testing, the experimenter identified the mice only by coded labels and was blinded to their group allocation. However, the investigator performing the subsequent data analysis was aware of the group allocation.

The pool was divided into four quadrants, and a hidden platform was positioned approximately 1 cm below the water surface in the target quadrant. During the training phase, each mouse was released sequentially from the four quadrants and allowed to search for the hidden platform. If a mouse failed to locate the platform within 60 s, it was gently guided to the platform and allowed to remain there for 30 s. Each mouse underwent four training trials per day for four consecutive days. During the probe test, the platform was removed, and each mouse was allowed to swim freely for 60 s. The total distance (within 60 s), distance in the target quadrant, number of entries into the target quadrant, time spent in the target quadrant and latency to first enter the target quadrant were recorded.

### 2.4. Immunohistochemistry

Immunohistochemical staining and image analysis were outsourced to Yilaibo Biotechnology Co., Ltd. (Shanghai, China). The tissue blocks were coded before being sent to the company, and the personnel responsible for sectioning, tissue processing, image acquisition, and image quantification were blinded to the genotype and treatment group of each mouse throughout the procedure. The staining protocol was adapted from previously described methods for evaluating Aβ deposition and glial activation in AD mouse models [13], with antibody-specific conditions optimized according to the manufacturers’ recommendations. After routine dehydration and paraffin embedding, coronal brain sections were prepared using a Leica RM2235 microtome. Following deparaffinization, the sections were incubated with 3% H_2_O_2_ (*v*/*v*) for 10 min to block endogenous peroxidase activity and then washed three times with PBS. Sections were subsequently incubated overnight at 4 °C with rabbit monoclonal anti-β-amyloid 1–42 antibody (ab201060, Abcam, Cambridge, UK), rabbit monoclonal anti-GFAP antibody (ab207165, Abcam), or rabbit monoclonal anti-Iba1 antibody (ab178846, Abcam). After washing, the sections were incubated with HRP-conjugated goat anti-rabbit IgG antibody (GB23303, Servicebio, Wuhan, China) at room temperature. Immunoreactivity was visualized using DAB chromogenic substrate (P0203, Beyotime Biotech. Inc., Shanghai, China), and the sections were mounted with neutral balsam. Images were captured using an ECLIPSE Ni microscope (Nikon Instruments Inc., Shanghai, China) at 200× magnification under identical imaging settings. For each marker (Aβ_1–42_, GFAP, or Iba-1), 3 mice per group were analyzed. For each mouse, 1 coronal section from a comparable anatomical level was evaluated, and 2 non-overlapping microscopic fields were captured within the predefined region of interest. The positively stained areas were quantified using Image Pro Plus software (version 6.0). The two field-level measurements were averaged to generate one value per mouse, and the mouse was treated as the biological experimental unit for statistical analysis.

### 2.5. Congo Red Staining

Congo red staining and image analysis were outsourced to Yilaibo Biotechnology Co., Ltd. (Shanghai, China). After paraffin embedding, the tissue blocks were coded and sent to the company, and the personnel responsible for sectioning, tissue processing, image acquisition, and image quantification were blinded to the genotype and treatment group of each mouse throughout the procedure. Congo red staining for amyloid deposits was performed using a standard histochemical procedure adapted from the classical Congo red method for amyloid detection [24]. Paraffin-embedded mouse brain sections were routinely deparaffinized, stained with Congo red, and mounted with neutral balsam. Images were captured under a microscope at 200× magnification using identical imaging settings for all groups. For Congo red staining, 3 mice per group were analyzed. For each mouse, 1 coronal section from a comparable anatomical level was evaluated, and 2 non-overlapping microscopic fields were captured within the predefined region of interest in the cerebral cortex or hippocampus. The integrated optical density (IOD) of Congo red-positive amyloid deposits was quantified separately in the cerebral cortex and hippocampus using Image-Pro Plus software (version 6.0). The two field-level measurements were averaged to generate one value per mouse, and the mouse was treated as the biological experimental unit for statistical analysis.

### 2.6. Hematoxylin and Eosin (H&E) Staining

Histological examination of colon tissue was outsourced to Yilaibo Biotechnology Co., Ltd. (Shanghai, China). After fixation in 10% formalin for 48 h, the tissue blocks were coded and sent to the company, and the personnel responsible for sectioning, tissue processing, and image acquisition were blinded to the genotype and treatment group of each mouse throughout the procedure. Histological examination of colon tissue was performed using a hematoxylin and eosin staining procedure adapted from a standard histological protocol [25]. After routine dehydration and paraffin embedding, tissues were sectioned at a thickness of 4–7 μm. Sections were subsequently stained with hematoxylin and eosin according to standard procedures. Images were captured using a microscope at 200 × magnification under identical imaging settings for all groups. For H&E staining, 3 mice per group were examined. For each mouse, 1 section from a comparable anatomical level was evaluated for morphological observation.

### 2.7. Enzyme-Linked Immunosorbent Assay (ELISA)

Neurotransmitter concentrations were determined using commercially available ELISA kits according to the manufacturers’ instructions. No modifications to the assay procedures specified by the manufacturers were introduced. The concentrations of glutamate (Glu), dopamine (DA) and γ-aminobutyric acid (GABA) in the brain tissue were measured using a Mouse Glu ELISA Kit (ml063299), Mouse DA ELISA Kit (ml002024) and Mouse GABA ELISA Kit (ml001894), respectively. The concentrations of 5-hydroxytryptamine (5-HT) in the brain tissue and whole blood were measured using Mouse 5-HT ELISA Kit (ml001891). All ELISA kits were purchased from Shanghai Enzyme-linked Biotechnology Co., Ltd. (Shanghai, China). Absorbance was measured using a SpectraMax M5 Multi-Mode Microplate Reader (Molecular Devices, San Jose, CA, USA), and analyte concentrations were calculated according to the corresponding kit instructions.

### 2.8. RNA Extraction and Quantitative Real-Time Polymerase Chain Reaction (PCR)

RNA extraction, cDNA synthesis, and quantitative real-time polymerase chain reaction were performed according to the corresponding manufacturers’ protocols, with the sample types and primer sets adapted to the objectives of the present study. Total RNA was extracted from colon tissue and whole blood samples with TRIzol reagent (15596026, Invitrogen, Carlsbad, CA, USA), and cDNA was synthesized by a Hifair^®^ II 1st Strand cDNA Synthesis SuperMix for qPCR (gDNA digester plus) (11123ES60, Yeasen Biotechnology, Shanghai, China). qPCR reactions were performed in a final volume of 20 μL on a QuantStudio 3 PCR system (Thermo Fisher Scientific, Waltham, MA, USA) using SYBR Green Master Mix (11202ES08, Yeasen Biotechnology). Relative gene expression was calculated using the 2^−ΔΔCt^ method as previously described [26]. All assays were repeated three times. The primer sequences used in this study are listed in Table 1.

### 2.9. Untargeted Metabolomics

Untargeted fecal metabolomics was performed by Shanghai Applied Protein Technology Co., Ltd. (Shanghai, China) using an established UHPLC–QTOF-MS workflow. Fecal samples from the Tg-PBS and Tg-KF7 groups were collected individually after 60 days of intervention and stored at −80 °C until analysis.

For metabolite extraction, approximately 80 mg of each frozen fecal sample was transferred on dry ice into a microcentrifuge tube. The sample was combined with 200 μL of H_2_O and five ceramic beads and homogenized. Subsequently, 800 μL of methanol/acetonitrile (1:1, *v*/*v*) was added for metabolite extraction. The mixture was centrifuged at 14,000× *g* for 20 min at 4 °C, and the resulting supernatant was dried in a vacuum centrifuge. For LC-MS analysis, dried extracts were reconstituted in 100 μL of acetonitrile/water (1:1, *v*/*v*), followed by centrifugation at 14,000× *g* for 15 min at 4 °C. The supernatant was subjected to LC-MS analysis using a 1290 Infinity UHPLC system (Agilent Technologies, Santa Clara, CA, USA) coupled to a TripleTOF 6600 quadrupole time-of-flight mass spectrometer (AB Sciex, Framingham, MA, USA).

After sum normalization, the processed metabolomics data were analyzed using the R package ropls (version 1.26.4). Multivariate analyses included Pareto-scaled principal component analysis (PCA) and orthogonal partial least-squares discriminant analysis (OPLS-DA). Seven-fold cross-validation and response permutation testing were used to evaluate model robustness. Variable importance in projection (VIP) scores were calculated from the OPLS-DA model to estimate the contribution of individual metabolites to group discrimination. Differences in individual metabolite abundance between the Tg-KF7 and Tg-PBS groups were assessed using independent-samples two-tailed *t*-tests. Metabolites with VIP > 1 and nominal *p* < 0.05 were defined as candidate differential metabolites for exploratory downstream analyses. To account for multiple testing, metabolite-level *p*-values were adjusted using the Benjamini–Hochberg false discovery rate (FDR) procedure, and FDR-adjusted *p* < 0.05 was considered statistically significant.

Candidate differential metabolites were mapped to pathways in the Kyoto Encyclopedia of Genes and Genomes (KEGG) database for pathway enrichment and pathway–metabolite network analyses. *p*-values from pathway enrichment analysis were adjusted for multiple testing using the Benjamini–Hochberg FDR procedure, with FDR-adjusted *p* < 0.05 considered statistically significant.

### 2.10. 16S rRNA Gene Sequence Analysis

16S rRNA gene sequencing and subsequent bioinformatics analyses were performed by Sinotech Genomics Co., Ltd. (Shanghai, China) according to the company’s standardized workflow, with the sample preparation and amplification conditions described below.

Mouse fecal samples were collected and immediately stored at −80 °C until DNA extraction. Genomic DNA was extracted from 30 mg of feces using the QIAamp DNA stool mini kit (Qiagen, Hilden, Germany) according to the manufacturer’s instructions, and DNA integrity was assessed by 1% agarose gel electrophoresis. The V3–V4 region of the bacterial 16S rRNA gene was amplified using the primers 338F (5′-ACTCCTACGGGAGGCAGCAG-3′) and 806R (5′-GGACTACHVGGGTWTCTAAT-3′). PCR amplification consisted of initial denaturation at 95 °C for 3 min, followed by 20 cycles of 95 °C for 30 s, 55 °C for 30 s, and 72 °C for 45 s, with a final extension at 72 °C for 10 min. PCR products were purified using an AxyPrep DNA Gel Extraction Kit (Axygen Biosciences, Union City, CA, USA), quantified with a Quantus™ Fluorometer (Promega, Madison, WI, USA), pooled at appropriate proportions, and used for library preparation with the NEXTFLEX^®^ Rapid DNA-Seq Kit (Bioo Scientific, Austin, TX, USA). Paired-end sequencing was performed on an Illumina MiSeq PE300 platform.

Raw paired-end reads were quality-filtered using Trimmomatic and merged using FLASH. Bases with quality scores below Q20 were trimmed using a 50-bp sliding window, and reads shorter than 50 bp after quality filtering were discarded. Paired-end reads were merged with a minimum overlap of 10 bp and a maximum mismatch ratio of 0.1. Samples were demultiplexed according to barcode and primer sequences, allowing no barcode mismatches and a maximum of two primer mismatches.

After quality filtering and read processing, a total of 1,280,138 valid sequences were obtained, with 33,717–50,307 sequences per sample (mean ± SD, 42,671 ± 3023 sequences/sample). Quality-filtered and merged sequences were clustered into operational taxonomic units (OTUs) at 97% sequence similarity using UPARSE version 7.1, with chimeric sequences removed during OTU clustering. Representative OTU sequences were taxonomically assigned using the RDP Classifier (version 2.11) against the SILVA reference database (Release 123), with a confidence threshold of 0.7. To minimize differences in sequencing depth among samples, the OTU abundance table was randomly rarefied to the sequencing depth of the sample with the lowest number of sequences before diversity analyses.

The sequencing data generated in this study have been deposited in an online repositories. The names of the repository/repositories and accession number(s) can be found below: https://www.ncbi.nlm.nih.gov, PRJNA1202228.

### 2.11. Statistical Analysis

During the 65-day experiment, two WT-PBS mice died on day 48 and one Tg-PBS mouse died on day 52. No necropsies were performed. For the longitudinal body-weight analysis, only mice with complete measurements at days 0, 30, and 60 were included in the repeated-measures ANOVA. Accordingly, body-weight measurements obtained before death from these three mice were not included in this longitudinal analysis. However, valid data collected before death for other analyses were retained where applicable. No data were excluded solely as statistical outliers. The sample size for each analysis is provided in the corresponding figure legend.

Body weight and MWM acquisition data were analyzed using mixed-design repeated-measures ANOVA, with time or training day as the within-subject factor and genotype and treatment as between-subject factors. Greenhouse–Geisser correction was applied when sphericity was violated. MWM probe-test outcomes, neurotransmitters, inflammatory cytokines, intestinal barrier-related genes, and Aβ_1–42_, GFAP, and Iba-1 immunohistochemical measurements were analyzed using two-way ANOVA with genotype and treatment as factors. Bonferroni-adjusted comparisons were performed where appropriate. For histological analyses, multiple microscopic fields were averaged to obtain one value per mouse. Congo red-positive amyloid deposition in Tg-PBS and Tg-KF7 mice was compared using an independent-samples two-tailed *t*-test.

Microbiota alpha diversity indices and taxonomic relative abundances were analyzed using the Kruskal–Wallis test, followed where appropriate by Dunn’s post hoc test. Benjamini–Hochberg FDR correction was applied to multiple comparisons. Beta-diversity was visualized by principal coordinate analysis based on unweighted UniFrac distances and assessed using pairwise ANOSIM with 999 permutations and FDR correction. Multivariate dispersion was evaluated separately by a permutation-based test.

For untargeted metabolomics, metabolites with VIP > 1 and nominal *p* < 0.05 were considered candidate differential metabolites for exploratory analysis. Metabolite- and KEGG pathway-level *p* values were adjusted using the Benjamini–Hochberg FDR procedure.

Spearman rank correlation analysis was used to examine associations of six selected bacterial genera with host phenotypic parameters and fecal metabolites. Benjamini–Hochberg FDR correction was applied separately to the genus–phenotype and genus–metabolite correlation sets.

Unless otherwise stated, data are presented as mean ± standard deviation (SD), and all tests were two-sided. Statistical significance was defined as *p* < 0.05 or FDR-adjusted *p* < 0.05, as appropriate.

## 3. Results

### 3.1. Effects of L. rhamnosus KF7-Fermented Milk on Spatial Learning and Memory in APP/PS1 Mice

**Figure 1:**
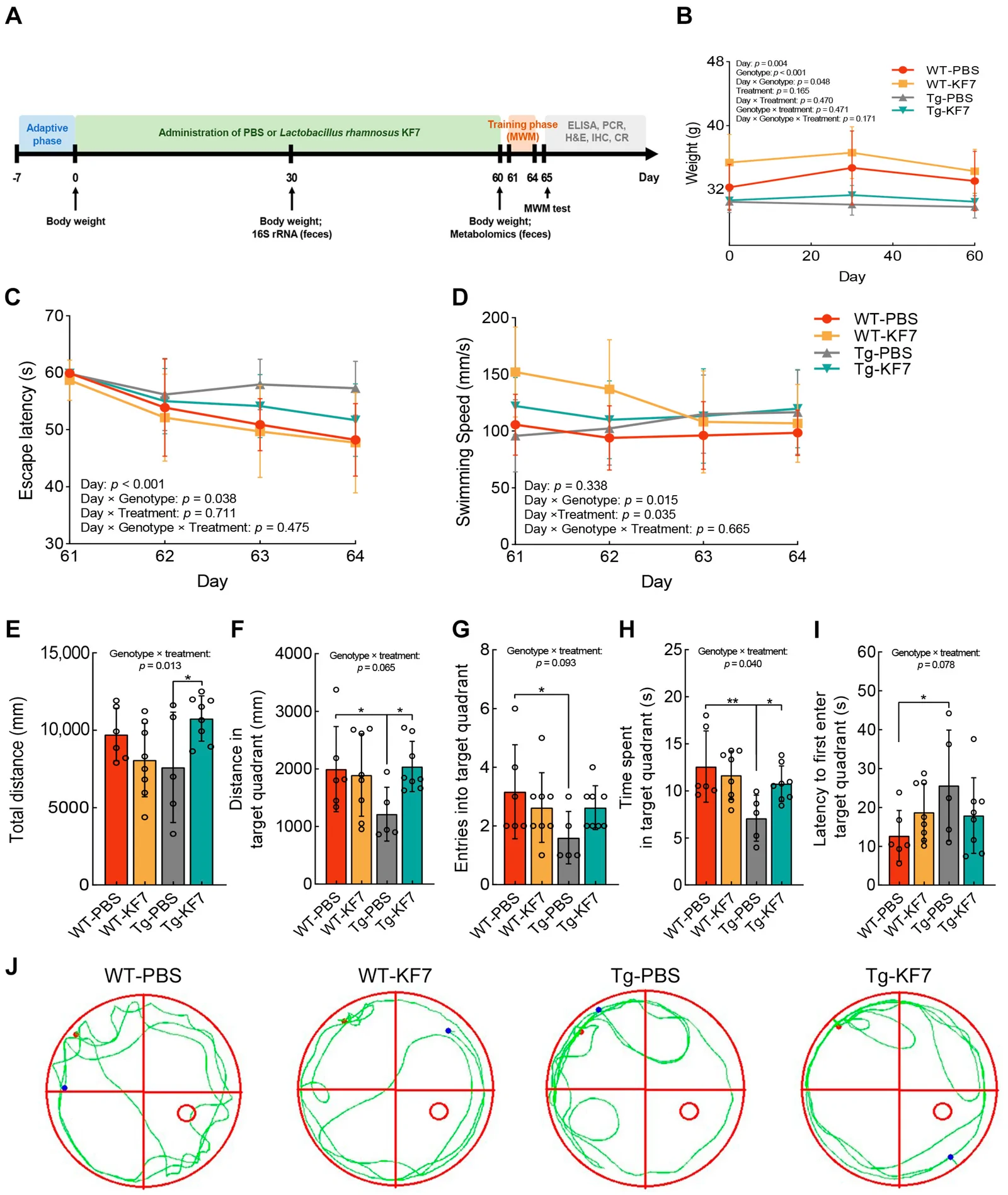
Effects of *L. rhamnosus* KF7-fermented milk on spatial learning and memory in APP/PS1 mice. (**A**) Experimental timeline. (**B**) Body weight changes. For the longitudinal body-weight analysis, only mice with complete measurements at days 0, 30, and 60 were included. Body-weight measurements obtained before death from mice that did not complete all three time points were not included in the repeated-measures analysis. Escape latency (**C**) and swimming speed (**D**) during the four-day Morris water maze (MWM) acquisition-training period. Total movement distance (**E**), movement distance in the target quadrant (**F**), number of entries into the target quadrant (**G**), time spent in the target quadrant (**H**) and latency to first enter the target quadrant (**I**) during the MWM probe test. (**J**) Representative swimming trajectories during the MWM probe test. WT-PBS, wild-type mice receiving PBS; WT-KF7, wild-type mice receiving KF7-fermented milk; Tg-PBS, APP/PS1 transgenic mice receiving PBS; Tg-KF7, APP/PS1 transgenic mice receiving KF7-fermented milk. WT-PBS n = 6; WT-KF7 n = 8; Tg-PBS n = 5; and Tg-KF7 n = 8. Data are presented as mean ± standard deviation. * *p* < 0.05, ** *p* < 0.01.

The experimental schedule is shown in Figure 1A. Body weight was measured before intervention (day 0), at mid-intervention (day 30) and after intervention (day 60). Body weight changed significantly over time (*p* = 0.004) and differed between genotypes (*p* < 0.001). However, no significant treatment-related effects on body weight were observed, including the time × treatment or time × genotype × treatment interactions (both *p* > 0.05), indicating that KF7-fermented milk did not significantly affect body weight during the experimental period (Figure 1B).

To evaluate the effects of KF7-fermented milk on cognitive function in APP/PS1 mice, MWM training and testing were performed from day 61 to day 65. During the four-day acquisition-training period, escape latency decreased significantly across training days (*p* < 0.001), with a significant main effect of genotype (*p* = 0.049) and a day × genotype interaction (*p* = 0.038). No significant effects of treatment, genotype × treatment, day × treatment, or day × genotype × treatment were observed (all *p* > 0.05). Swimming speed showed no significant main effects of day, genotype, or treatment (all *p* > 0.05), but significant day × genotype and day × treatment interactions were detected (*p* = 0.015 and *p* = 0.035, respectively). The day × genotype × treatment interaction was not significant (*p* = 0.665) (Figure 1C,D).

During the probe test, Tg-PBS mice showed a shorter movement distance in the target quadrant (*p* = 0.045), fewer entries into the target quadrant (*p* = 0.033), and less time spent in the target quadrant (*p* = 0.003) than WT-PBS mice, together with a longer latency to first enter the target quadrant (*p* = 0.034). No significant difference in total movement distance was observed between the two PBS-treated groups (*p* = 0.140). Significant genotype × treatment interactions were observed for total movement distance (*p* = 0.013) and time spent in the target quadrant (*p* = 0.040). In Tg mice, KF7-fermented milk treatment was associated with greater total movement distance, greater target-quadrant movement distance, and longer time spent in the target quadrant than PBS treatment (*p* = 0.024, 0.026, and 0.026, respectively), while target-quadrant entries and entry latency did not differ significantly between treatments (*p* > 0.05) (Figure 1E–I). These results suggest that KF7-fermented milk partially reduced the differences observed between Tg-PBS and WT-PBS mice in selected MWM probe-test measures. Representative swimming trajectories are shown in Figure 1J.

### 3.2. Effects of L. rhamnosus KF7-Fermented Milk on Amyloid Plaque Deposition and Neurotransmitter Concentrations in APP/PS1 Mice

**Figure 2:**
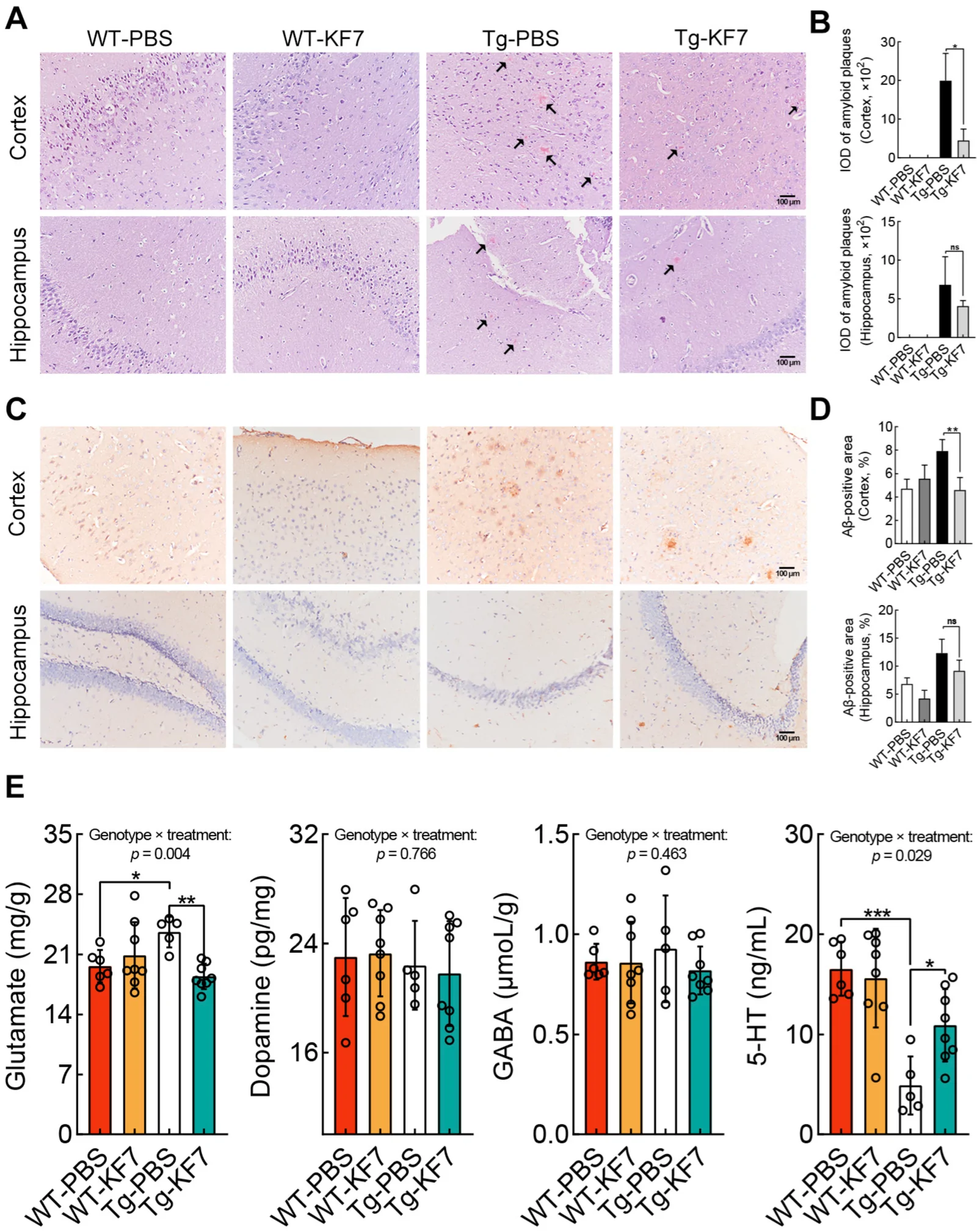
Effects of *L. rhamnosus* KF7-fermented milk on amyloid plaque deposition and neuro-transmitter concentrations in APP/PS1 mice. (**A**) Congo red staining of amyloid plaques in the cerebral cortexes and hippocampus. Congo red-positive plaques are indicated by black arrows. (**B**) Quantitative analysis of Congo red-positive amyloid plaques in the cerebral cortexes and hippocampus. (**C**) Immunohistochemical staining of Aβ_1–42_ deposition (brown plaques) in the cerebral cortexes and hippocampus. (**D**) Quantitative analysis of the Aβ_1–42_-positive area in the cerebral cortex and hippocampus. (**E**) Concentrations of glutamate (Glu), dopamine (DA), and γ-aminobutyric acid (GABA) in brain tissue and 5-hydroxytryptamine (5-HT) in whole blood. WT-PBS, wild-type mice receiving PBS; WT-KF7, wild-type mice receiving KF7-fermented milk; Tg-PBS, APP/PS1 transgenic mice receiving PBS; Tg-KF7, APP/PS1 transgenic mice receiving KF7-fermented milk. For Congo red staining (**A**,**B**) and immunohistochemical analyses (**C**,**D**), WT-PBS, WT-KF7, Tg-PBS, and Tg-KF7 groups contained n = 3 mice per group. For neurotransmitter analysis (**E**), WT-PBS, WT-KF7, Tg-PBS, and Tg-KF7 groups contained n = 6, 8, 5, and 8 mice, respectively. Data are presented as mean ± standard deviation. ns, not significant; * *p* < 0.05, ** *p* < 0.01, *** *p* < 0.001.

Congo red staining showed amyloid plaques in the cerebral cortex and hippocampus of eight-month-old APP/PS1 mice, whereas no positive staining was detected in WT mice. In Tg mice, KF7-fermented milk was associated with a lower amyloid plaque burden in the cerebral cortex than PBS treatment (*p* = 0.025), whereas no significant difference was observed in the hippocampus (*p* = 0.270) (Figure 2A,B). Aβ is thought to be an initial pathological abnormality in AD, and Aβ_1–42_ is among the most abundantly generated Aβ isoforms [27]. As shown in Figure 2C,D, Aβ_1–42_ immunohistochemistry showed strong immunoreactivity in the cerebral cortex of Tg-PBS mice. A significant genotype × treatment interaction was detected in the cerebral cortex (*p* = 0.007), and Tg-KF7 mice showed a lower Aβ_1–42_ plaque burden than Tg-PBS mice (*p* = 0.004). In the hippocampus, the difference between Tg-KF7 and Tg-PBS mice was not statistically significant (*p* = 0.067).

Neurotransmitter analysis revealed a significant genotype × treatment interaction for brain Glu concentrations (*p* = 0.004). Tg-KF7 mice had lower Glu concentrations than Tg-PBS mice (*p* = 0.002), whereas no significant treatment-related difference was observed in WT mice (*p* = 0.374). No significant genotype × treatment interactions or treatment-related differences were observed for DA or GABA. A significant genotype × treatment interaction was also detected for whole-blood 5-HT concentrations (*p* = 0.029). Tg-PBS mice had lower 5-HT concentrations than WT-PBS mice (*p* < 0.001), whereas Tg-KF7 mice had higher concentrations than Tg-PBS mice (*p* = 0.011). No treatment-related difference was observed in WT mice (*p* = 0.649), and no significant difference in brain 5-HT was detected (Figure 2E). Overall, KF7-fermented milk was associated with lower cortical amyloid plaque burden, lower brain Glu concentrations, and higher whole-blood 5-HT concentrations in APP/PS1 mice.

### 3.3. Effects of L. rhamnosus KF7-Fermented Milk on Neuroinflammation and Inflammatory Responses in APP/PS1 Mice

**Figure 3:**
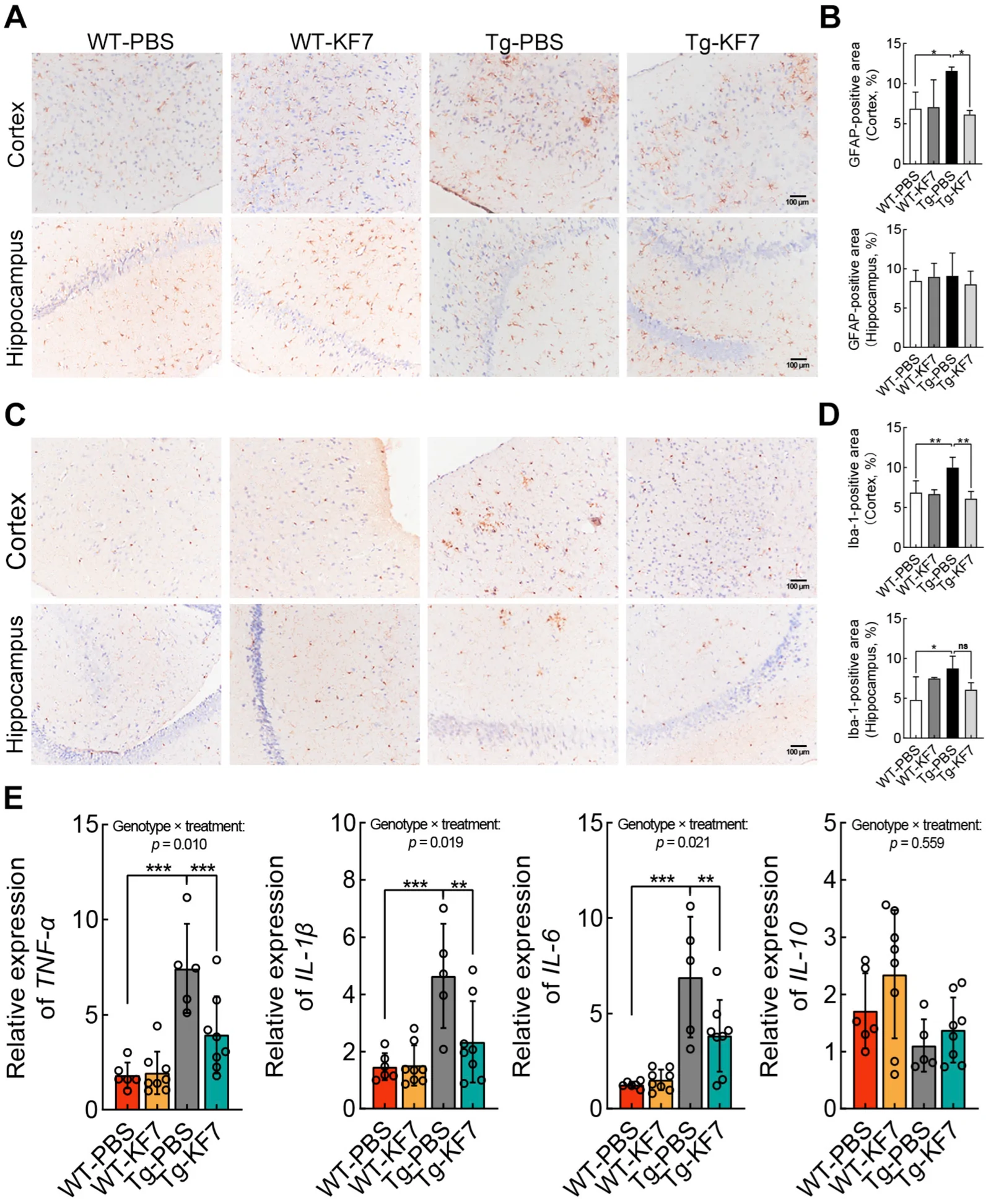
Effects of *L. rhamnosus* KF7-fermented milk on neuroinflammation and inflammatory responses in APP/PS1 mice. (**A**) Immunohistochemical staining of GFAP in the cerebral cortexes and hippocampus. (**B**) Quantitative analysis of GFAP-positive area in the cerebral cortexes and hippocampus. (**C**) Immunohistochemical staining of Iba-1 in the cerebral cortexes and hippocampus. (**D**) Quantitative analysis of Iba-1-positive area in the cerebral cortexes and hippocampus. (**E**) Relative mRNA expression levels of the inflammatory cytokines *TNF-α*, *IL-1β*, *IL-6*, and *IL-10* in whole blood. WT-PBS, wild-type mice receiving PBS; WT-KF7, wild-type mice receiving KF7-fermented milk; Tg-PBS, APP/PS1 transgenic mice receiving PBS; Tg-KF7, APP/PS1 transgenic mice receiving KF7-fermented milk. For immunohistochemical analysis (**A**–**D**), WT-PBS, WT-KF7, Tg-PBS, and Tg-KF7 groups contained n = 3 mice per group. For inflammatory cytokine mRNA analysis (**E**), WT-PBS, WT-KF7, Tg-PBS, and Tg-KF7 groups contained n = 6, 8, 5, and 8 mice, respectively. Data are presented as mean ± standard deviation. ns, not significant; * *p* < 0.05, ** *p* < 0.01, *** *p* < 0.001.

To evaluate neuroinflammatory responses, astrocyte and microglial activation were assessed by GFAP and Iba-1 immunohistochemical staining in the cerebral cortex and hippocampus. As shown in Figure 3A–D, GFAP and Iba-1 immunoreactivity was increased in the cerebral cortex of Tg-PBS mice compared with WT-PBS mice. Significant genotype × treatment interactions were observed for cortical GFAP (*p* = 0.044) and Iba-1 (*p* = 0.020). KF7-fermented milk reduced GFAP- and Iba-1-positive areas in the cortex of Tg mice (*p* = 0.012 and *p* = 0.003, respectively), whereas no significant treatment-related differences were observed in WT mice. In the hippocampus, no significant differences were detected for GFAP. Although a significant genotype × treatment interaction was detected for hippocampal Iba-1 (*p* = 0.026), the reduction in Tg-KF7 mice compared with Tg-PBS mice did not reach statistical significance (*p* = 0.093).

Inflammatory cytokine mRNA levels in whole blood were further analyzed by PCR. *TNF-α*, *IL-1β*, and *IL-6* levels were higher in Tg-PBS mice than in WT-PBS mice (all *p* < 0.001). Significant genotype × treatment interactions were observed for *TNF-α*, *IL-1β*, and *IL-6* (*p* = 0.010, 0.019, and 0.021, respectively). Tg-KF7 mice showed lower *TNF-α*, *IL-1β*, and *IL-6* levels than Tg-PBS mice, whereas no significant treatment-related differences were observed in WT mice. *IL-10* showed a significant genotype effect (*p* = 0.017), but no significant treatment effect or genotype × treatment interaction was detected (Figure 3E). These findings indicate genotype-dependent responses to KF7-fermented milk, with reduced cortical glial activation and lower whole-blood pro-inflammatory cytokine expression observed in APP/PS1 mice.

### 3.4. Effects of L. rhamnosus KF7-Fermented Milk on Colonic Histology and Intestinal Barrier-Related Gene Expression in APP/PS1 Mice

**Figure 4:**
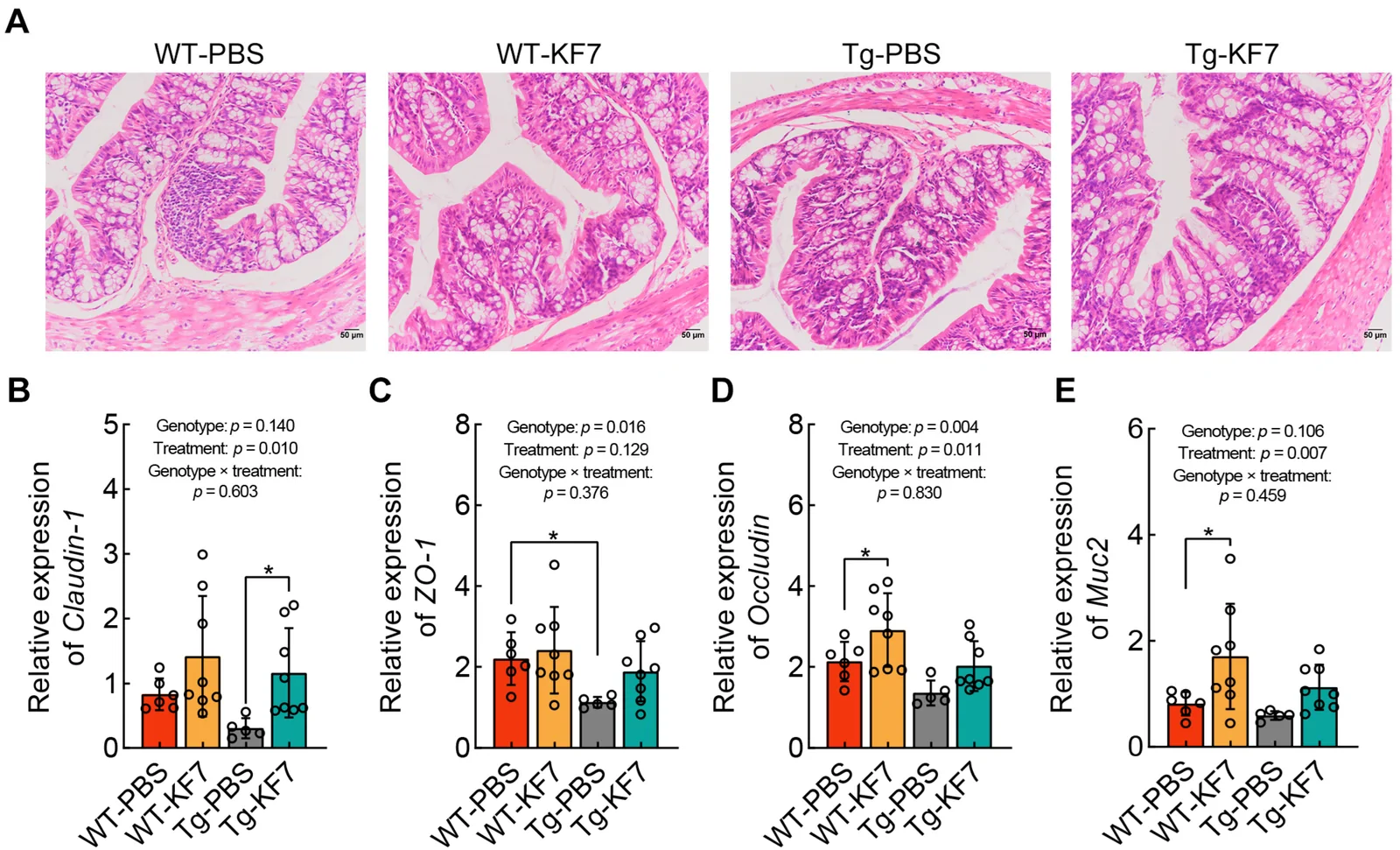
Effects of *L. rhamnosus* KF7-fermented milk on colonic histology and intestinal barrier-related gene expression in APP/PS1 mice. (**A**) Representative H&E staining of mouse colonic tissue. Relative mRNA expression of the intestinal barrier-related genes *Claudin-1* (**B**), *ZO-1* (**C**), *Occludin* (**D**), and *MUC-2* (**E**) in the colon. WT-PBS, wild-type mice receiving PBS; WT-KF7, wild-type mice receiving KF7-fermented milk; Tg-PBS, APP/PS1 transgenic mice receiving PBS; Tg-KF7, APP/PS1 transgenic mice receiving KF7-fermented milk. For H&E staining (**A**), WT-PBS, WT-KF7, Tg-PBS, and Tg-KF7 groups contained n = 3 mice per group. For intestinal barrier-related gene expression analysis (**B**–**E**), WT-PBS, WT-KF7, Tg-PBS, and Tg-KF7 groups contained n = 6, 8, 5, and 8 mice, respectively. Data are presented as mean ± standard deviation. * *p* < 0.05.

H&E staining showed generally preserved colonic architecture in all groups, with intact epithelial lining and regularly arranged crypts (Figure 4A). No obvious epithelial erosion, crypt distortion, inflammatory cell infiltration, or submucosal edema was observed.

Despite the absence of overt histopathological changes, differences in intestinal barrier-related gene expression were detected (Figure 4B–E). *ZO-1* expression was significantly lower in Tg-PBS mice than in WT-PBS mice (*p* = 0.033). Significant treatment effects were observed for *Claudin-1*, *Occludin*, and *MUC-2* (*p* = 0.010, 0.011, and 0.007, respectively), with no significant genotype × treatment interactions. Bonferroni-adjusted comparisons showed that KF7-fermented milk increased *Claudin-1* expression in Tg mice (*p* = 0.031) and increased *Occludin* and *MUC-2* expression in WT mice (*p* = 0.040 and 0.012, respectively). These findings indicate alterations in intestinal barrier-related gene expression in APP/PS1 mice and suggest that KF7-fermented milk modulates the expression of selected barrier-associated genes.

### 3.5. Effects of L. rhamnosus KF7-Fermented Milk on Gut Microbiota in APP/PS1 Mice

**Figure 5:**
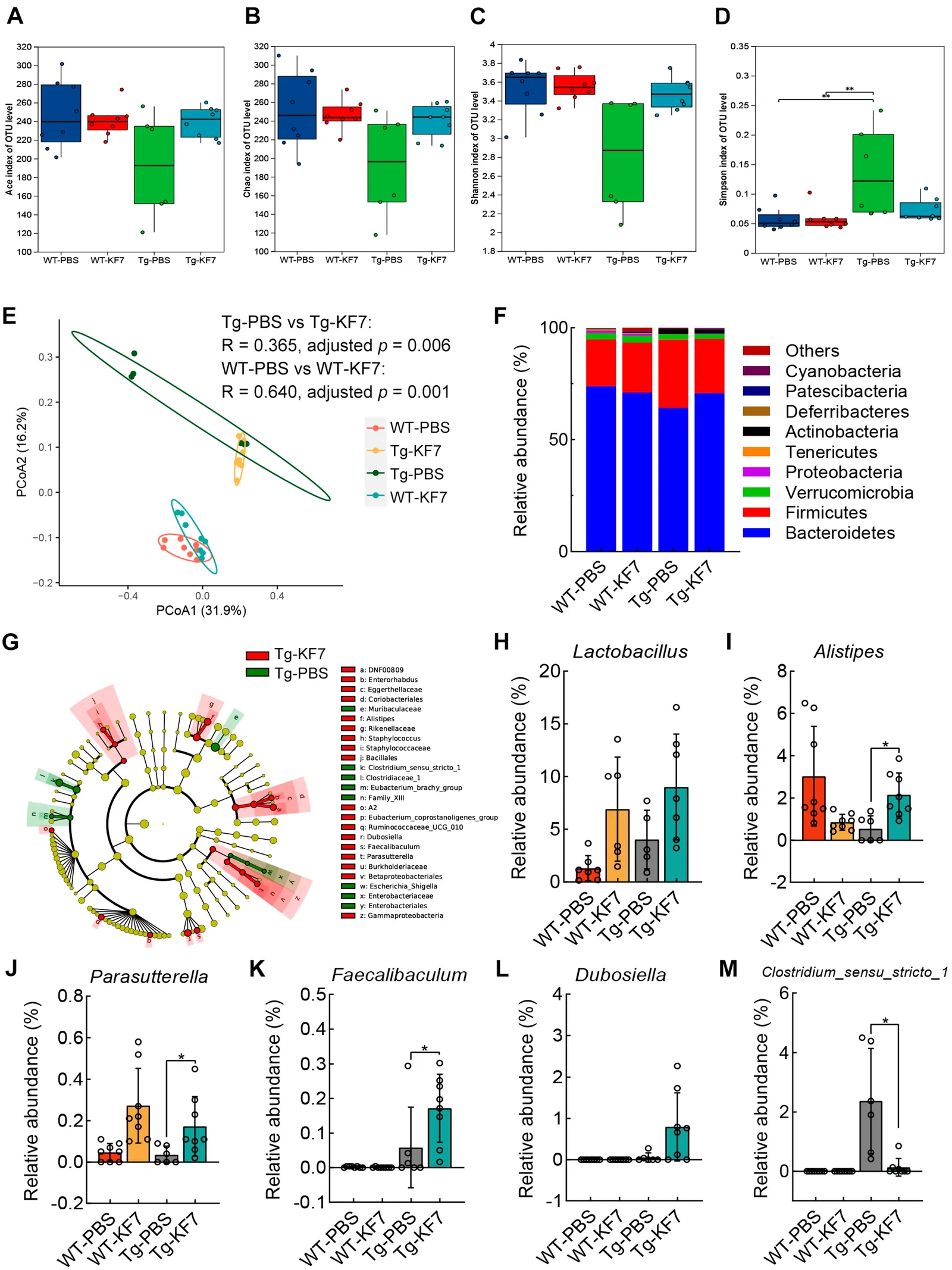
Effects of *L. rhamnosus* KF7-fermented milk on gut microbiota in APP/PS1 mice. (**A**–**D**) Alpha diversity of the gut microbiota assessed using the ACE, Chao, Shannon, and Simpson indices. (**E**) Principal coordinate analysis (PCoA) of gut microbial communities based on unweighted UniFrac distances. (**F**) Relative abundance of gut microbiota at the phylum level. (**G**) Linear discriminant analysis Effect Size (LEfSe) was conducted to analyze differences in relative taxonomic abundance between the two groups (red area: Tg-KF7, green area: Tg-PBS) from the phylum to the genus level (LDA value > 2, *p* < 0.05). Red and green nodes indicate microbial taxa enriched in the Tg-KF7 and Tg-PBS groups, respectively, whereas yellow nodes indicate taxa without significant differences between the two groups. Relative abundance of (**H**) *Lactobacillus*, (**I**) *Alistipes*, (**J**) *Parasutterella*, (**K**) *Faecalibaculum*, (**L**) *Dubosiella*, and (**M**) *Clostridium_sensu_stricto_1* at the genus level. WT-PBS, wild-type mice receiving PBS; WT-KF7, wild-type mice receiving KF7-fermented milk; Tg-PBS, APP/PS1 transgenic mice receiving PBS; Tg-KF7, APP/PS1 transgenic mice receiving KF7-fermented milk. WT-PBS n = 8; WT-KF7 n = 8; Tg-PBS n = 6; and Tg-KF7 n = 8. Data are expressed as mean ± standard deviation. * FDR-adjusted *p* < 0.05, ** FDR-adjusted *p* < 0.01.

As shown in Figure 5A–D, no significant overall differences were detected among the four groups for the ACE or Chao indices after FDR correction (FDR-adjusted *p* > 0.05). The Shannon index was lower in Tg-PBS mice and tended to shift toward WT levels following KF7-fermented milk intervention, although the overall difference was not significant after FDR correction (FDR-adjusted *p* = 0.086). In contrast, the Simpson index differed significantly among groups (FDR-adjusted *p* = 0.022), with a higher value in Tg-PBS mice and a shift toward WT levels in Tg-KF7 mice. These findings suggest altered alpha diversity in APP/PS1 mice, particularly in community evenness, with partial normalization associated with KF7-fermented milk intervention.

Principal coordinates analysis (PCoA) based on unweighted UniFrac distances showed differences in gut microbial community structure among the groups (Figure 5E). Significant pairwise differences were detected between Tg-PBS and Tg-KF7 (R = 0.365, FDR-adjusted *p* = 0.006), Tg-KF7 and WT-KF7 (R = 0.996, FDR-adjusted *p* = 0.001), Tg-PBS and WT-PBS (R = 0.580, FDR-adjusted *p* = 0.001), and WT-KF7 and WT-PBS mice (R = 0.640, FDR-adjusted *p* = 0.001). However, multivariate dispersion also differed significantly among groups, indicating that within-group variability may have contributed to the observed beta-diversity differences.

At the phylum level (Figure 5F and Table 2), *Bacteroidetes* and *Firmicutes* were the dominant bacteria in the gut microbiota, with the relative abundance of the two bacteria combined reaching more than 90%. Significant overall differences were observed for *Bacteroidetes*, *Firmicutes*, *Proteobacteria*, *Tenericutes*, and *Actinobacteria* after FDR correction (overall FDR-adjusted *p* < 0.05). Pairwise comparisons showed lower *Tenericutes* abundance in Tg-PBS than in WT-PBS mice (FDR-adjusted *p* = 0.026) and in Tg-KF7 than in WT-PBS and WT-KF7 mice (FDR-adjusted *p* = 0.010 and 0.029, respectively). *Actinobacteria* abundance was higher in Tg-KF7 than in WT-PBS mice (FDR-adjusted *p* = 0.026). No significant phylum-level differences were detected between Tg-KF7 and Tg-PBS mice after FDR correction. Detailed pairwise comparisons are provided in Supplementary Table S1. Overall, these findings indicate genotype- and treatment-associated differences in gut microbial diversity and community structure, whereas treatment-related changes at the phylum level were limited.

At the genus level (Figure 5H–M and Supplementary Table S2), the Tg-KF7 group showed significantly higher relative abundances of *Alistipes*, *Parasutterella*, and *Faecalibaculum* and a lower abundance of *Clostridium_sensu_stricto_1* than the Tg-PBS group (all FDR-adjusted *p* < 0.05). *Dubosiella* showed a marked numerical increase in Tg-KF7 mice, but the difference did not remain significant after FDR correction (FDR-adjusted *p* = 0.075). No significant difference in *Lactobacillus* abundance was detected.

LEfSe analysis of the Tg-PBS and Tg-KF7 groups identified several discriminative taxa (Figure 5G). At the genus level, *Alistipes*, *Parasutterella*, *Faecalibaculum*, and *Dubosiella* were enriched in Tg-KF7 mice, whereas *Clostridium_sensu_stricto_1* was enriched in Tg-PBS mice. As LEfSe was used as an exploratory analysis, inferential conclusions were based primarily on the FDR-adjusted comparisons described above. Overall, these results indicate that KF7-fermented milk was associated with selective genus-level alterations in the gut microbiota of APP/PS1 mice.

### 3.6. Effects of L. rhamnosus KF7-Fermented Milk on Fecal Metabolic Profiles in APP/PS1 Mice

**Figure 6:**
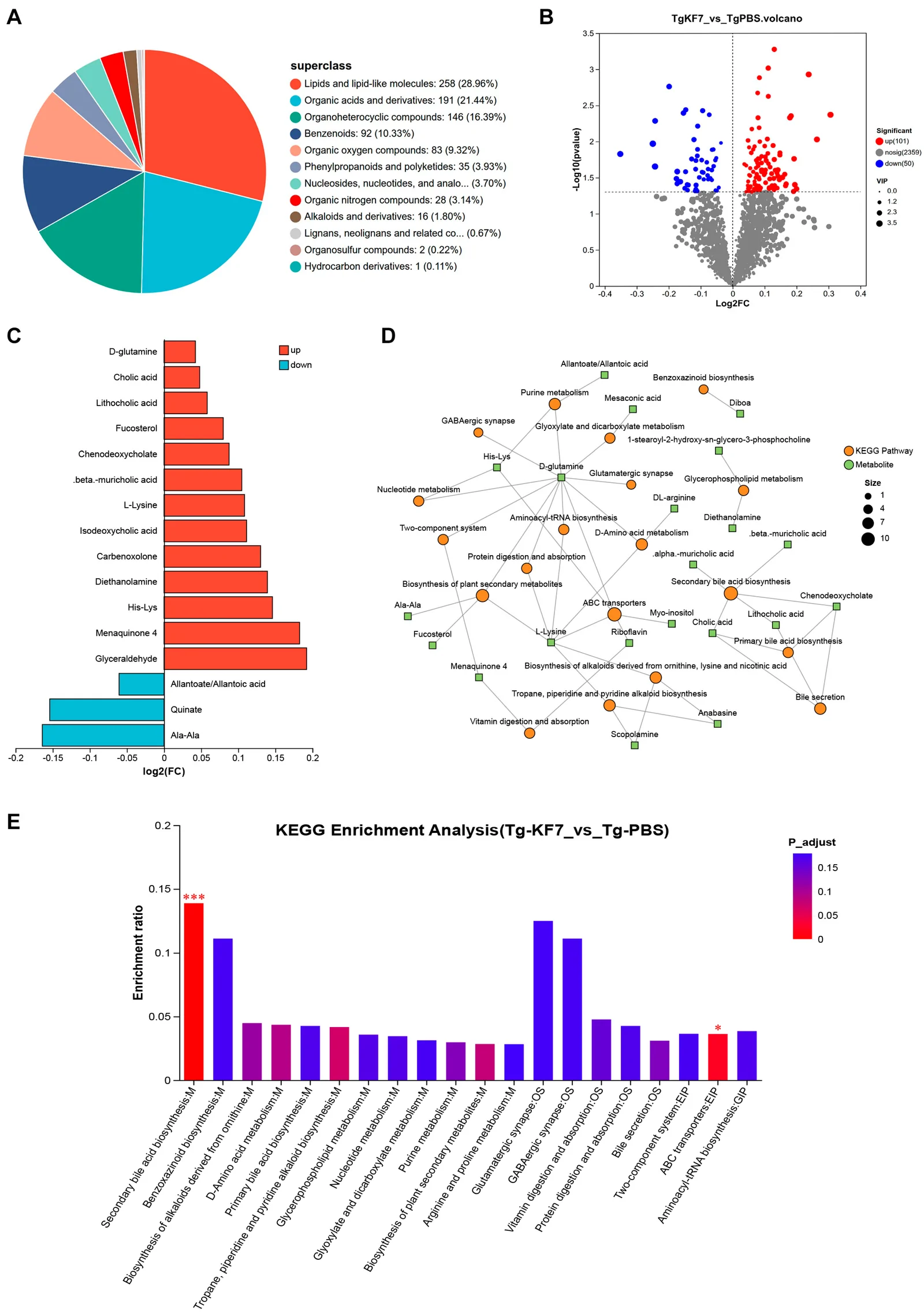
Effects of *L. rhamnosus* KF7-fermented milk on fecal metabolic profiles in APP/PS1 mice. (**A**) Classification and relative proportions of the detected fecal metabolites. (**B**) Volcano plot comparing fecal metabolite profiles between the Tg-KF7 and Tg-PBS groups. Candidate metabolites were identified using the exploratory criteria of VIP > 1 and nominal *p* < 0.05; red and blue indicate metabolites with increased and decreased abundance, respectively, in the Tg-KF7 group, whereas gray indicates metabolites that did not meet these criteria. (**C**) Fold changes of candidate metabolites meeting the exploratory screening criteria, with red and blue indicating increased and decreased abundance, respectively, in the Tg-KF7 group relative to the Tg-PBS group. None of the individual metabolite differences remained significant after FDR correction. (**D**) KEGG pathway–metabolite network based on the candidate metabolites. Green squares represent metabolites and orange circles represent KEGG pathways; pathway node size is proportional to the number of mapped candidate metabolites. (**E**) KEGG pathway enrichment analysis of candidate metabolites. Enrichment *p*-values were adjusted for multiple testing using the Benjamini–Hochberg false discovery rate (FDR) procedure, and the color gradient indicates FDR-adjusted statistical significance. * FDR-adjusted *p* < 0.05, *** FDR-adjusted *p* < 0.001. WT-PBS, wild-type mice receiving PBS; WT-KF7, wild-type mice receiving KF7-fermented milk; Tg-PBS, APP/PS1 transgenic mice receiving PBS; Tg-KF7, APP/PS1 transgenic mice receiving KF7-fermented milk. Tg-PBS n = 5; and Tg-KF7 n = 8. The data were analyzed using the Majorbio Cloud Platform, https://www.majorbio.com/tools (accessed on 17 January 2026).

Untargeted metabolomic profiling was performed on fecal samples from the Tg-PBS and Tg-KF7 groups collected at the end of the intervention. Lipids and lipid-like molecules, organic acids and derivatives, organoheterocyclic compounds, and benzenoids were the major metabolite classes detected, accounting for 28.96%, 21.44%, 16.39%, and 10.33% of the identified metabolites, respectively (Figure 6A).

Using VIP > 1 and nominal *p* < 0.05 as predefined exploratory screening criteria, 151 candidate differential metabolites were identified between the Tg-KF7 and Tg-PBS groups, including 101 with increased and 50 with decreased abundance in Tg-KF7 mice. Several bile acid-related metabolites, including β-muricholic acid, isodeoxycholic acid, cholic acid, and lithocholic acid, showed higher abundance in the Tg-KF7 group. However, none of the individual metabolites remained significant after FDR correction (FDR-adjusted *p* > 0.05); therefore, these metabolite-level differences were considered exploratory (Figure 6B,C).

KEGG pathway enrichment analysis showed that secondary bile acid biosynthesis was the most significantly enriched pathway and remained significant after FDR correction (FDR-adjusted *p* = 5.687 × 10^−5^). ABC transporters were also significantly enriched (FDR-adjusted *p* = 0.020), whereas glutamatergic synapse, GABAergic synapse, and benzoxazinoid biosynthesis did not remain significant after correction. Network analysis further linked secondary bile acid biosynthesis to several bile acid-related candidate metabolites, whereas D-glutamine was associated with multiple pathways, including glutamatergic and GABAergic synapses. Because the corresponding metabolite-level differences were not significant after FDR correction, these associations were considered exploratory (Figure 6D,E).

Overall, fecal metabolomic profiling identified secondary bile acid biosynthesis as the strongest FDR-supported metabolic pathway associated with KF7-fermented milk intervention in APP/PS1 mice. These findings reflect fecal metabolic associations and do not provide direct evidence of altered host signaling or gut–brain communication.

### 3.7. Associations Between Gut Microbiota, Fecal Metabolites, and AD-Related Indices

**Figure 7:**
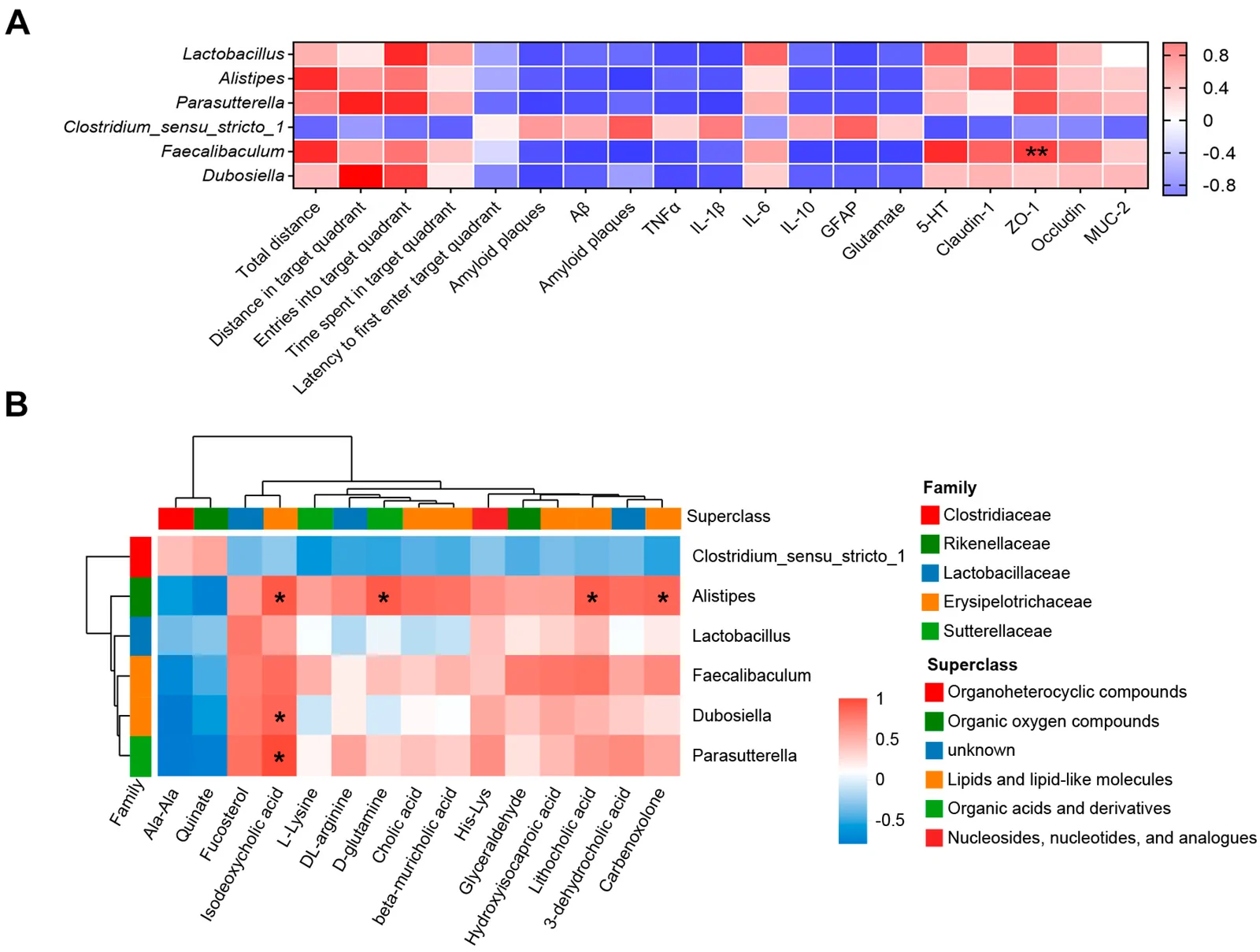
Associations between gut microbiota, fecal metabolites, and AD-related indices. (**A**) Spearman correlation analysis between bacterial genera and cognitive behavioral or biochemical indices. The color scale represents Spearman correlation coefficients. Nominal *p*-values were adjusted for multiple testing using the Benjamini–Hochberg false discovery rate (FDR) procedure across the correlations shown in panel (**A**). (**B**) Spearman correlation analysis between bacterial genera and fecal metabolites. Red and blue indicate positive and negative correlations, respectively, with color intensity representing correlation strength. Nominal *p*-values were adjusted for multiple testing using the Benjamini–Hochberg FDR procedure across the correlations shown in panel (**B**). * FDR-adjusted *p* < 0.05, ** FDR-adjusted *p* < 0.01. WT-PBS, wild-type mice receiving PBS; WT-KF7, wild-type mice receiving KF7-fermented milk; Tg-PBS, APP/PS1 transgenic mice receiving PBS; Tg-KF7, APP/PS1 transgenic mice receiving KF7-fermented milk. Tg-PBS n = 5; and Tg-KF7 n = 8. The data were analyzed using the Majorbio Cloud Platform, https://www.majorbio.com/tools (accessed on 17 January 2026).

Spearman correlation analysis was performed to examine associations between bacterial genera and AD-related behavioral, biochemical, and metabolic measures. As showed in Figure 7A and Supplementary Table S3, several associations between bacterial genera and behavioral or biochemical indices reached nominal *p* < 0.05, but only the positive correlation between *Faecalibaculum* abundance and ZO-1 expression remained significant after FDR correction (FDR-adjusted *p* = 0.003). All other associations were considered exploratory.

Microbe–metabolite correlation analysis identified several FDR-supported associations (Figure 7B and Supplementary Table S4). Isodeoxycholic acid was positively associated with *Parasutterella* (FDR-adjusted *p* = 0.014), *Alistipes* (FDR-adjusted *p* = 0.034), and *Dubosiella* (FDR-adjusted *p* = 0.046). *Alistipes* was also positively associated with D-glutamine (FDR-adjusted *p* = 0.034), lithocholic acid (FDR-adjusted *p* = 0.038), and carbenoxolone (FDR-adjusted *p* = 0.046). Overall, these findings identify isodeoxycholic acid as a metabolite showing robust associations with several bacterial genera and suggest that *Alistipes* is associated with a broader set of metabolomic alterations. Nevertheless, because the microbiome and metabolomic datasets were obtained at different sampling time points, these microbe–metabolite associations should be interpreted as exploratory.

## 4. Discussion

APP/PS1 mice reproduce major AD-like features, including amyloid deposition, proliferation of neuroglial cells, and cognitive impairment [28,29]. In the present study, APP/PS1 mice showed impaired spatial memory together with cortical and hippocampal amyloid pathology. KF7-fermented milk was associated with better probe-test performance and lower amyloid burden, particularly in the cortex. Similar effects have been reported after microbiota-targeted interventions: fecal microbiota transplantation from wild-type donors improved Morris water maze performance in APP/PS1 mice [30], while long-term consumption of Tibetan fermented milk improved learning and memory in the same model [31].

Other *L. rhamnosus*-containing products have also shown beneficial effects on cognition, although the strains, delivery formats, and experimental models differ substantially. For example, several *L. rhamnosus* strains have attenuated cognitive deficits and inflammatory responses in models induced by Aβ, scopolamine, noise, or chronic stress [32,33,34,35,36]. Of particular relevance, *L. rhamnosus* CBT-LR5 administered with skim milk alleviated scopolamine-induced cognitive impairment and improved intestinal barrier and inflammatory markers in mice [37], while a 12-week randomized trial reported cognitive benefits of the same strain–milk combination in older adults with mild cognitive impairment [38]. These findings support the potential cognitive relevance of *L. rhamnosus*-containing dairy products, but direct comparison with the present study is limited because none of these studies used a transgenic AD model or directly compared *L. rhamnosus*-fermented milk with conventional fermented milk.

The fermentation substrate may also influence functional outcomes. Fermentation of different cereal substrates with *L. rhamnosus* has been shown to produce distinct metabolite profiles and cognitive effects [39], suggesting that strain–substrate interactions are important. Skim milk was used in the present study because of its established application in previous *L. rhamnosus* studies [19,20,37], consistent composition, and suitability for fermentation. However, there is currently insufficient evidence to identify an optimal milk matrix for AD-related applications, and no direct comparison has established whether *L. rhamnosus*-fermented milk is more effective than conventional fermented milk. Future studies incorporating unfermented milk, conventional fermented milk, and different milk matrices will be required to distinguish the contributions of the bacterial strain, milk matrix, and fermentation-derived components.

Aβ accumulation is considered an early and central pathological feature of AD and is closely linked to disease progression and cognitive dysfunction. Previous studies have shown that Aβ plaque deposition is age-dependent and often appears first in the cerebral cortex before becoming more widespread in later disease stages [40,41,42]. Our histological observations are consistent with this pattern, as the cortical changes were more pronounced than those in the hippocampus. The reduction in cortical Congo red and Aβ_1–42_ staining is consistent with evidence linking gut microbiota modulation to amyloid pathology. Germ-free APP/PS1 mice showed 31–42% lower cerebral Aβ_42_ levels and 57–77% lower compact plaque load than colonized controls [43], while encapsulated *Lactiplantibacillus plantarum* reduced brain Aβ_1–42_ from 2.537 to 2.185 µg/mL in APP/PS1 mice [44]. Although additional protein-level validation would be needed to confirm the present changes more quantitatively, our histological findings are consistent with this body of evidence.

KF7-fermented milk was also associated with lower brain glutamate and higher whole-blood 5-HT in transgenic mice, whereas DA, GABA, and brain 5-HT were unchanged. Excess extracellular glutamate contributes to excitotoxicity and may promote Aβ-related neuronal dysfunction [45,46,47,48]. Peripheral 5-HT is largely produced by intestinal enterochromaffin cells and is strongly influenced by the gut microbiota [49,50,51,52]. In mice, gut bacteria have been estimated to determine approximately 64% of colonic and 49% of serum 5-HT [53], and colonic 5-HT concentrations increased from 17 ± 3 ng/mg in germ-free mice to 25 ± 2 ng/mg in mice colonized with a human microbiota [54]. Thus, the increase in whole-blood 5-HT observed here is compatible with microbiota-related regulation of peripheral serotonin. The absence of a corresponding change in brain 5-HT suggests that peripheral and central serotonergic responses should be interpreted separately.

Neuroinflammation, including microgliosis and astrogliosis, has been reported to be closely related to AD [55]. Activated microglia and astrocytes can amplify inflammatory signaling and promote neuronal dysfunction in response to amyloid pathology [56,57]. In this study, KF7-fermented milk reduced elevated GFAP and Iba-1 immunoreactivity in the cerebral cortex of APP/PS1 mice, indicating attenuated cortical glial activation. Gut microbiota markedly contribute to glial activation: germ-free APPPS1 mice showed 64% and 40% less cortical Iba-1 immunostaining than colonized controls at 3.5 and 8 months, respectively [43], and certain probiotics reduced IBA-1-positive microglia in 5 × FAD mice [58]. KF7-fermented milk also lowered whole-blood *TNF-α*, *IL-1β*, and *IL-6* mRNA levels; *IL-10* showed a genotype effect but no treatment effect. Similarly, encapsulated *L. plantarum* decreased brain TNF-α and IL-1β immunoreactivity in APP/PS1 mice [44], and *L. rhamnosus* CBT-LR5 with skim milk lowered serum TNF-α in a cognitive impairment model [37]. These strain-specific findings emphasize that probiotic effects cannot be extrapolated between *L. rhamnosus* strains. These findings support the possibility that KF7-fermented milk may modulate both systemic and brain-related inflammatory responses in APP/PS1 mice.

Colonic morphology remained largely intact, but molecular differences in barrier-related genes were evident. *ZO-1* expression was lower in Tg-PBS mice than in WT-PBS mice, while KF7-fermented milk was associated with higher *Claudin-1* expression in transgenic mice and higher *Occludin* and *MUC-2* expression in wild-type mice. Similar reductions in ZO-1, occludin, and claudin-1, together with increased intestinal permeability, have been reported in APP_swe_/PS1_ΔE9_ mice [59]. These results suggest that molecular alterations in the intestinal barrier may precede overt histological damage. Nevertheless, the absence of significant genotype × treatment interactions for several genes indicates that the barrier-related effects should be interpreted cautiously.

Gut microbial composition also differed between Tg-KF7 and Tg-PBS mice. In transgenic mice, the Simpson index shifted toward wild-type levels after intervention, whereas the Shannon index showed a similar but non-significant trend. *Alistipes*, *Parasutterella*, and *Faecalibaculum* were more abundant in Tg-KF7 mice, while *Clostridium_sensu_stricto_1* was less abundant; the increase in *Dubosiella* did not remain significant after FDR correction. Several of these taxa have been linked to bile acid metabolism, epithelial homeostasis, or inflammatory regulation. *Alistipes*, a producer of succinate as well as acetate and propionate [60], has also been associated with bile acid-related metabolic alterations [61,62], suggesting a potential role in modulating bile acid signaling and host inflammatory homeostasis. *Parasutterella*, a core member of the gut microbiota in humans and mice, has been implicated in bile acid maintenance and cholesterol metabolism [63]. *Faecalibaculum rodentium* can affect epithelial homeostasis by increasing epithelial turnover, crypt prolif-eration, and MHC II expression [64]. *Dubosiella* has been reported to show anti-inflammatory effects, with its abundance positively related to IL-10 and negatively related to TNF-α, IL-6, and IL-1β [65]. In contrast, *Clostridium_sensu_stricto_1* has been associated with inflammation-related genes [66]. These changes therefore suggest that KF7-fermented milk was associated with a distinct microbial community profile, although the lack of baseline microbiome measurements prevents direct assessment of within-animal microbial changes.

Untargeted metabolomics suggested parallel alterations in bile acid-related metabolism. β-muricholic acid, isodeoxycholic acid, cholic acid, and lithocholic acid were higher among the exploratory candidate metabolites in Tg-KF7 mice, but none remained significant after metabolite-level FDR correction. In contrast, secondary bile acid biosynthesis and ABC transporter pathways remained significantly enriched after FDR correction. Altered bile acid metabolism has been reported in AD, and several bile acids can influence inflammatory signaling through receptors such as FXR and TGR5 [67,68,69,70]. The present metabolite-level findings should therefore be regarded as hypothesis-generating, while the pathway-level results provide stronger evidence for an association between KF7-fermented milk and bile acid-related metabolism. D-glutamine and glutamatergic/GABAergic pathways were also identified as exploratory features, but fecal metabolites should not be interpreted as direct measures of brain metabolism.

Correlation analyses provided additional exploratory links between microbial and host features. Only the positive association between *Faecalibaculum* and ZO-1 remained significant after FDR correction, whereas *Alistipes* showed several associations with bile acid-related metabolites and D-glutamine. Because microbiota and metabolomics samples were collected at different time points, these correlations cannot demonstrate coordinated microbiota–metabolite mechanisms or causality. They should instead be considered hypothesis-generating observations for future strain-level, depletion, or transplantation studies.

An important limitation is the use of PBS rather than unfermented milk as the control. Because the intervention simultaneously introduced the milk matrix, viable KF7 cells, and fermentation-derived compounds, the observed effects should be attributed to KF7-fermented milk as a whole product rather than specifically to the *L. rhamnosus* KF7 strain. Appropriate unfermented milk and fermentation controls will be needed to distinguish these contributions in future studies.

## 5. Conclusions

KF7-fermented milk was associated with better cognitive performance, lower cortical amyloid burden, reduced inflammatory indices, differences in intestinal barrier-related gene expression, and shifts in gut microbial and fecal metabolic profiles in APP/PS1 mice. The microbiota and metabolomics findings further suggest possible involvement of bile acid-related pathways, although most individual metabolite and correlation signals were exploratory.

These findings should be interpreted as effects of KF7-fermented milk as a whole-product intervention rather than effects attributable specifically to the KF7 strain. The lack of baseline microbiome and metabolomic measurements, temporally unmatched multi-omics sampling, and inclusion of male mice only further limit longitudinal and mechanistic interpretation. Future studies with appropriate dairy controls, longitudinal sampling, strain-level tracking, both sexes, and temporally matched multi-omics analyses are warranted.

## Figures and Tables

**Table 1:** Sequences of primers used for PCR.

Gene Name	Sequence	
*Claudin-1*	forward	5′-GGCTTCTCTGGGATGGATCG-3′
	reverse	5′-CCCCAGCAGGATGCCAATTA-3′
*ZO-1*	forward	5′-ATCCCACAAGGAGCCATTCC-3′
	reverse	5′-TAGGGTCACAGTGTGGCAAG-3′
*Occludin*	forward	5′-AGCAATGACATGTATGGCGGA-3′
	reverse	5′-CCTCTGTCCCAAGCAAGTGT-3′
*MUC-2*	forward	5′-GAAGCCAGATCCCGAAACCA-3′
	reverse	5′-GAATCGGTAGACATCGCCGT-3′
*TNF-α*	forward	5′-AGGCACTCCCCCAAAAGATG-3′
	reverse	5′-TTGCTACGACGTGGGCTAC-3′
*IL-6*	forward	5′-CAACGATGATGCACTTGCAGA-3′
	reverse	5′-TGGAAATTGGGGTAGGAAGGAC-3′
*IL-1β*	forward	5′-ATGCCACCTTTTGACAGTGATG-3′
	reverse	5′-AGCTTCTCCACAGCCACAAT-3′
*IL-10*	forward	5′-GGTTGCCAAGCCTTATCGGA-3′
	reverse	5′-GACACCTTGGTCTTGGAGCTTA-3′
*GAPDH*	forward	5′-CTGCCCAGAACATCATCC-3′
	reverse	5′-CTCAGATGCCTGCTTCAC-3′

**Table 2:** Relative abundance of gut microbiota in mice at the phylum level.

Phylum	WT-PBS	WT-KF7	Tg-PBS	Tg-KF7	Overall FDR-Adjusted *p*
*Bacteroidetes*	73.88 ± 6.84% ^a^	71.08 ± 8.10% ^a^	64.19 ± 5.98% ^a^	59.09 ± 13.01% ^a^	0.045
*Firmicutes*	20.91 ± 6.95% ^a^	22.39 ± 6.98% ^a^	30.50 ± 8.29% ^a^	34.00 ± 11.18% ^a^	0.043
*Proteobacteria*	1.13 ± 1.88% ^a^	0.96 ± 1.04% ^a^	0.12 ± 0.12% ^a^	0.24 ± 0.12% ^a^	0.041
*Tenericutes*	0.48 ± 0.39% ^a^	0.29 ± 0.25% ^ab^	0.04 ± 0.06% ^bc^	0.01 ± 0.01% ^c^	0.001
*Actinobacteria*	0.43 ± 0.39% ^a^	0.50 ± 0.27% ^ab^	2.33 ± 3.20% ^ab^	2.93 ± 2.73% ^b^	0.041

Data are expressed as mean ± standard deviation (%). WT-PBS, wild-type mice receiving PBS; WT-KF7, wild-type mice receiving KF7-fermented milk; Tg-PBS, APP/PS1 transgenic mice receiving PBS; Tg-KF7, APP/PS1 transgenic mice receiving KF7-fermented milk. WT-PBS, n = 8; WT-KF7, n = 8; Tg-PBS, n = 6; Tg-KF7, n = 8. Overall differences among the four groups were evaluated using the Kruskal–Wallis test with Benjamini–Hochberg false discovery rate (FDR) correction. Pairwise comparisons were performed using Dunn’s post hoc test with FDR correction. Different superscript letters within the same row indicate significant pairwise differences (FDR-adjusted *p* < 0.05); groups sharing at least one letter are not significantly different.

## Data Availability

The datasets presented in this study can be found in online repositories. The names of the repository/repositories and accession number(s) can be found below: https://www.ncbi.nlm.nih.gov, PRJNA1202228. Data will be made available on request.

## Supplementary Materials

The following supporting information can be downloaded at https://www.mdpi.com/article/10.3390/foods15183304/s1, Table S1: Statistical comparisons of phylum-level relative abundances among experimental groups; Table S2: Genus-level differential abundance statistics for bacterial taxa; Table S3: Targeted Spearman correlation analysis between six bacterial genera and host phenotypic parameters; Table S4: Targeted genus-metabolite Spearman correlations.
